# Microscopical Memoranda

**Published:** 1874-05-01

**Authors:** 


### Microscopical Memoranda.

COLLATED BY LESTER CURTIS, M.D.

HAIR, IN ITS MICROSCOPICAL AND MEDICO-LEGAL ASPECTS.—Dr. E. Hofman, in the December number of *The Lens*, calls attention to this important but too much neglected subject.

A question of the greatest moment may arise as to whether certain hairs are from brutes or men, and, if from men, from what part of the body they come.

He first describes the well-known appearance of a hair, which consists of three layers, the outer, or cuticular layer, composed of one layer of cells lying over the other, like tiles on a roof; the second, or cortical portion, consisting of closely packed spindle-cells, which can be separated by the action of dilute sulphuric acid; and the third, or central medullary portion, sometimes absent. The cortical substance contains the coloring matter of the hair, diffused among its cells. Cavities filled with air are found in it, especially in dry hair, and in that of old persons; these cavities are not found in the hair of young persons.

The medullary portion, when well developed, is about one-fourth the diameter of the hair. Dr. Hofman considers this substance to be made up of cells, although there is a difference of opinion on the point; it is frequently interrupted, and contains *no* pigment, the supposed pigment-granules being minute air-bubbles. The medullary substance is often wanting, particularly in blonde hair; it is never present in woolly hair, or in the hair of the new-born child; it is more constant in hair from other parts of the body than in that from the head.

In medico-legal cases, the question might arise whose the hair was; and from what part of the body it came. The first question must be decided by comparing both the gross and microscopical appearance with that of the person concerned. In deciding to what part of the body the hairs belong, the length, the size, the form, and the root of the hair, must be noticed. The hair from the head and beard are less limited in length than the hair from other portions of the body, although circumstances may modify the length. The size of the hair differs in different parts of the body, and may form a diagnostic mark. The beard is the thickest, generally measuring 0.14 to 0.15 mm. (.056 to .059 inch); next comes the hair on the female genitals, 0.15 mm.; then the hair of the eyebrows, 0.12 mm.

(.047 inch); the hair about the male genitals, 0.11 mm. (.043 inch); finally, the hair from the head, in either sex, 0.06 to 0.08 mm. (.023 to .031 inch). The other individual differences may render the value of the size for diagnosis less reliable; and the same hair may vary in diameter in different parts.

Hair of the head is generally round; but when it is curly it is flattened; the transverse section is then oval. The beard is generally triangular, on section, with one convex side. Hair from the genitals is generally oval, sometimes, however, it is triangular, with one convex side. Hair which has been exposed to sweat is sometimes swollen in one part, and so changed in form.

When the hair grows undisturbed, it ends in a fine point; all the hair of a young child is of this kind; so, too, the hair which begins to grow at puberty. This may be a guide as to the age of a person. Hair which has been cut has, at first, a sharply-defined transverse section; it afterwards becomes rounded and smaller, or frayed out. This may point to the time which has elapsed since the hair was cut. The beard, being less frequently cut, is more often split and frayed out. The hair of the female is also generally frayed at the ends.

The shape taken by the ends of the hair depends upon the action of friction and sweat, the former splitting and rubbing off the ends, the latter dissolving the connective substance. The sweat changes the color of the hair, as in the axilla, on the scrotum, and labia

The hair of animals usually differs greatly from that of man, though preserving the same general structure. The cuticula has, in most animals, absolutely and relatively larger cells. The medullary substance differs greatly from that in human hair, the cellular structure being usually evident without any reagent.

---

THE DETECTION OF BLOOD BY THE SPECTRUM MICROSCOPE.—In a recent number of THE EXAMINER we described a method of detecting blood by the use of an alcoholic solution of guiacum, and an ethereal solution of peroxid of hydrogen (ozonic ether). Sometimes, however, the amount of blood is so minute that this process will not be applicable; in such cases the spectrum microscope may be used, as described by Mr. Sorbey, in Guy's Hospital Reports for 1869-70. If there be not too little, he soaks a small piece of the stained fabric in a few drops of water, in a watch-glass; he then squeezes out the liquid, and allows it to stand for a short time in the glass, in order to deposit any insoluble matter, and then pours it into the cell which he uses for examining spectra. These cells are made of an ordinary piece of barometer tubing, having an internal diameter of one-eighth to one-tenth of an inch, and a length of one-half to three-quarters of an inch. One end of this tube is connected to a slip of glass, like an ordinary cell for examining liquids. If the stain which is being examined is a fresh one, it shows the spectrum of fresh blood, which has two well-defined lines in the green. If, however, the blood has been exposed some time to the action of the air, these lines will be fainter, and another would be seen in the red. The relative distinctness of these lines shows the age of the stain; but in forming the conclusion it is necessary to know the circumstances; for the sulphurous acid in the air of towns will cause more change in one day than the pure air of the country will in a week.

In order to make the result more certain, it is well to examine the spectrum after the action of the reagents. If a bit of citric acid, about one-fiftieth of an inch in diameter, is added to the fluid in the cell, the bands will disappear, and will not be restored by addition of ammonia. Scarcely any other coloring matter will be effected in this way. After the addition of the citric acid, and the *excess* of the ammonia, if a piece of sulphate of protoxide of iron, not above one-hundredth of an inch in diameter, be added to the cell, a very characteristic spectrum of deoxidized hæmatin will be seen. This spectrum shows an absorption-band in the green, with a second fainter band near the blue end. It is necessary, in obtaining these results, to avoid exposing the substances in the cell for any length of time, to the air, by filling the cell full, and cementing a thin glass cover on its top with marine glue.

If the blood falls on leather, or any substance containing tannic acid, it may be impossible to detect the coloring matter by this method, although present in considerable quantity, as the tannic acid precipitates the coloring matter. Sometimes, however, by cutting a thin shaving of the leather, and dissolving off the blood, avoiding as much as possible wetting the leather, and then proceeding as before, blood may be obtained which has not been acted on by the tannin, and the spectrum seen. By this method he claims to be able to detect as little as one-thousandth of a grain of blood.

He also uses the same method for the detection of blood in urine, except that he uses a tube one-fourth of an inch in diameter and ten inches long. A drop of blood in a pint of urine will give a distinct spectrum.

---

PSEUDO-MUSCULAR HYPERTROPHY.--The *Philadelphia Medical Times* contains a translation of an article on this subject. After describing the usual symptoms and course of the disease, and the microscopic appearance as being simply an atrophy of the muscular fibre, accompanied by an enormous increase of the interstitial fatty and connective tissue, the writer passes on to the consideration of four cases, which, though presenting some points of similarity, were in others markedly different from the ordinary course of the disease. In each of these cases the disease was consequent upon an injury. The functional derangements were not so marked as in typical cases: in place of being totally lost, the power of motion was only diminished. The microscope revealed, in each case, what appeared to be a true hypertrophy of the muscular fibres, without excessive growth of the interstitial connective tissue. From this it would seem that the hypertrophy spreads from the muscle to the connective tissue, and the hypertrophied connective tissue, pressing on the muscle, causes atrophy afterwards. Schlesinger, however, reports a case of a man with mental disease, in whom some of the muscles were hypertrophied. The microscope showed the muscular tissue much diseased, but in them there was no hypertrophy of the muscular fibre. Whether the process is a simple inflammation, or what is its nature, is not known.

---

EPISCLERAL MELANOTIC SARCOMA.—Dr. H. C. Markham reports, in the *Medical Record*, a case of a melanotic sarcoma occurring as a primary tumor, and growing from the "upper and inner margin of the cornea. Aside from its history and color, it bore a resemblance to a pterygium." It was confined to the sclerotic entirely. An attempt was made to dissect it out; but as it returned persistently, the eyeball was enuncleated. Subsequent to the operation, a "small bluish tumor, about the size of a grain of corn, and situated on the inferior surface of the supraorbital plate," appeared. This was removed, and the surrounding tissues thoroughly cauterized. After a year the tumor had not re-appeared.

The only other case of the kind known to the writer, was one reported by D. H. Knapp, of New York city.

---

FATTY DEGENERATION OF THE HEART IN WOMEN DYING SUDDENLY AFTER DELIVERY.—Dr. Philipps reports five cases of sudden death in women, soon after delivery, in none of whom had there been the loss of more than a small quantity of blood. Fatty degeneration of the heart was found in each of the cases.—*Schmidt's Jahr.*

